# Understanding the cancer stem cell

**DOI:** 10.1038/sj.bjc.6605821

**Published:** 2010-07-27

**Authors:** S Bomken, K Fišer, O Heidenreich, J Vormoor

**Affiliations:** 1 Northern Institute for Cancer Research, Paul O’Gorman Building, Newcastle University, Framlington Place, Newcastle Upon Tyne, NE2 4HH, UK; 2 Department of Paediatric Oncology, Royal Victoria Infirmary, Queen Victoria Road, Newcastle Upon Tyne, NE1 4LP, UK

**Keywords:** cancer stem cell, leukaemia stem cell, self-renewal, clonal evolution, tumour heterogeneity

## Abstract

The last 15 years has seen an explosion of interest in the cancer stem cell (CSC). Although it was initially believed that only a rare population of stem cells are able to undergo self-renewing divisions and differentiate to form all populations within a malignancy, a recent work has shown that these cells may not be as rare as thought first, at least in some malignancies. Improved experimental models are beginning to uncover a less rigid structure to CSC biology, in which the concepts of functional plasticity and clonal evolution must be incorporated into the traditional models. Slowly the genetic programmes and biological processes underlying stem cell biology are being elucidated, opening the door to the development of drugs targeting the CSC. The aim of ongoing research to understand CSCs is to develop novel stem cell-directed treatments, which will reduce therapy resistance, relapse and the toxicity associated with current, non-selective agents.

Over the last 15 years, major advances have been made in identifying the malignant population responsible for tumour maintenance and initiation of relapse. Many names have been used to identify this population but the term cancer stem cell (CSC) has received broad acceptance. Cancer stem cells have been defined as ‘a cell within a tumor that possess (*sic*) the capacity to self-renew and to cause the heterogeneous lineages of cancer cells that comprise the tumor’ (Clarke *et al*, 2006). These two definitive biological properties are what make the CSC the prime candidate for initiation of relapse, thereby becoming a crucial target for the development of novel therapies (Figure 1).

Although accurately portraying the ability of this population to self-renew and populate an entire tissue, albeit a malignant one, the term CSC has led to some confusion. The CSC is commonly assumed to have developed from a normal tissue stem cell and, as such, thought to be the cell from which a malignancy originated. There is an ongoing debate over whether CSCs represent a mature tissue stem cell which has undergone malignant change or whether more differentiated cells re-initiate a ‘stemness’ programme as part of, or following, malignant transformation. This question will need to be answered for each malignancy in turn. Until we have this information, it is important to consider independently the concepts of cell of origin and cancer-propagating stem cell, as defined purely by self-renewal and capacity to differentiate.

In this minireview, we will consider the history of CSC research, the successes achieved so far and the translational importance of understanding CSC biology. The original studies in CSC biology were undertaken by John Dick in Toronto, who identified a hierarchy of stem cell potential, which mimicked the normal haematopoietic stem cell (HSCs) hierarchy. It is because this work laid the foundations for all subsequent CSC research, as well as our detailed understanding of normal haematopoiesis, that many of the global lessons within this subject are taken from haematological malignancies. Nevertheless, these lessons are, frequently, equally pertinent to solid tumour stem cell biology and we will endeavour to show the parallels in this review.

## The history of CSC research

That many malignancies, both solid and haematological, show significant physical heterogeneity has been known for many years, indeed since the earliest pathological assessments were undertaken. More recently this physical heterogeneity has been complemented by increasing awareness of variation in both molecular and functional biology, as assessed by *in vitro* and *in vivo* assays. These differences have driven the search for the population within a heterogeneous malignancy, which is able to maintain the disease, and crucially, initiate relapse once clinical remission has been achieved. Experimentally, this population is identified by its ability to serially repopulate a malignancy, either *in vitro* or *in vivo*.

The notion that only a small proportion of a malignant population might be able to transfer a tumour developed during early transplantation experiments. These have recently been discussed extensively by Dick (2008). Furth and Kahn (1937) inoculated inbred mice with single cells derived from a leukaemia arising in the same inbred strain. They identified that only a small number, approximately 5%, of inoculations resulted in successful transplantation. Solid tumour transplantation work, conducted throughout the 1950's, intended primarily to answer the question of whether malignancy was a virally transmitted phenomenon. Retrospectively, these studies provided further evidence that although single-cell inoculation could initiate a malignancy in a recipient animal, this was achieved on only a minority of occasions (Ishibashi, 1950; Hewitt, 1953; Makino, 1956).

During the 1960's the spleen colony-forming assay, developed by James Till and Ernest McCulloch, was first used to accurately enumerate the proportion of murine lymphoma cells capable of colony formation *in vivo* (Bruce and Van Der Gaag, 1963). This work again showed that colony formation was restricted to approximately 1% of transplanted cells. Furthermore, the path was set for the development of what we now recognise as the concept of the CSC. Splenic colonies, each of which they presumed had developed from a single malignant cell, were able to transplant lymphoma on to a second generation of recipient mice. These early serial transplantations suggested that a small proportion of malignant cells were able to self-renew to give rise to a very large number of malignant progeny. However, more recent studies have shown that this is not the case across all malignancies (Kelly *et al*, 2007; Quintana *et al*, 2008; le Viseur *et al*, 2008).

## Models of tumour heterogeneity and their place in the CSC model

**Figure 2:**
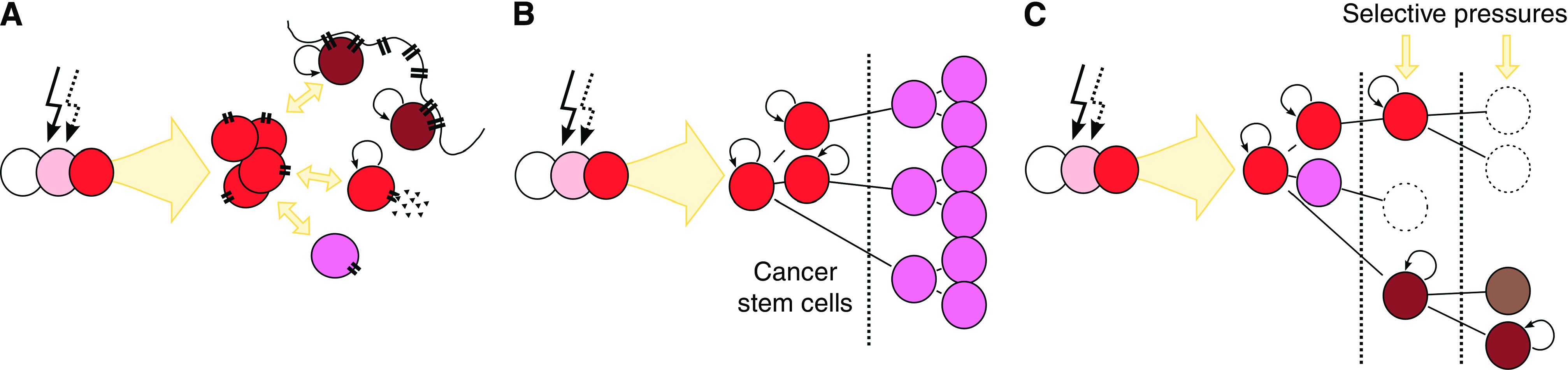
After oncogenesis, induced by an initial event (solid arrow) with or without supporting events (hashed arrow), cells differentiate to form a heterogeneous tumour. Two models have been proposed to explain this (**A** and **B**). The process of clonal evolution (**C**) is likely to underlie the ongoing development of certain tumour characteristics such as drug resistance. (**A**) Stochastic model. Variations in phenotype and biology result from intrinsic and extrinsic factors including niche interactions (=) and intercellular signalling (▴). These signals may be available to any cell at a particular time, with the correct combination of factors able to initiate the CSC programme, and therefore self-renewal (curved arrows) in any member of the population. (**B**) Hierarchy model. A tumour shows a hierarchy analogous to the normal tissue hierarchy, with a restricted pool of cells showing self-renewal (curved arrows) and differentiation potential. Differentiated tumour cells form the bulk of the tumour mass but are unable to self-renew. (**C**) Clonal evolution. An ongoing process, beginning before the clinical presentation, wherein sequential genetic and epigenetic changes result in a polyclonal population with differing survival potential under the selective pressure of therapy. Clonal evolution may be seen within tumours following either the stochastic or hierarchy model.

Tumour heterogeneity could now be shown at the functional level in addition to the morphological level. Given a presumed single cell of origin for any individual malignancy, the basis for this functional heterogeneity has been explained by one of two models. The stochastic model predicts that a malignancy is composed of a homogeneous population of cells, which generate their heterogeneity in response to particular combinations of endogenous and exogenous factors. Endogenously these would include gene dosage effects, transcriptional and translational control mechanisms, whereas exogenously cytokine concentrations, cell–cell interactions and particularly niche environment would all be important (Figure 2A). The hierarchy model predicts that a malignancy is organised in a manner analogous to the normal tissue hierarchy with cancer/tissue stem cells able to produce identical daughter stem cells with self-renewal capacity, and committed progenitor daughter cells with limited, although potentially still significant, potential to divide. With greater differentiation, so reproductive potential diminishes (Figure 2B).

It has recently been argued by Shackleton *et al* (2009) that the hierarchy model, with a rare CSC at the apex, is essentially synonymous with the CSC model. Heterogeneity in malignancies not fitting this model results from a random process of genetic changes and selective advantage. They further argue that the increasing frequency of tumour-propagating cells in the most sensitive modern assays, shows that we should avoid trying to fit all malignancies to the CSC model. Although it is true to say that CSC theory may not be applicable to all malignancies, it may be equally true that not all CSCs fit the hierarchy model. Indeed, both the hierarchy and stochastic models are compatible with CSC theory. In the stochastic model, stemness exists as a functional phenotype, which could be shown by any member of the malignant population given the appropriate endogenous and exogenous factors. Most plausibly, having occupied a suitable niche, a cell now able to express its self-renewal programme and producing daughter cells which differentiate to populate the bulk malignancy, becomes a CSC. The stochastic model does not yet predict whether stemness is found truly within each population, or whether cells first undergo a process of de-differentiation to a more tissue-specific stem cell-like phenotype, reacquiring stemness in the process. This plasticity within a cell lineage, between the CSC and non-CSC compartments, is known as bi-directional interconvertibility (Gupta *et al*, 2009). It makes no prediction of stem-cell frequency but simply argues that with the correct signals, the stem cell programme can be activated in otherwise non-CSC malignant cells. In this way it is distinct from the concept of lineage plasticity, in which, for example, lymphoid to myeloid change may be seen. Of course, neither one of these models need necessarily be the only correct model, and it is quite plausible that different malignancies might fit one or other theory.

Recently, the CSC field has been trying to accommodate a further biological phenomenon into its models. There is now convincing evidence that cancer cells, stem cells included, are subject to a process known as clonal evolution. In clonal evolution, new clones continuously develop, emerging with new genetic, and potentially epigenetic, changes. Environmental pressures result in constantly adapting cancer cell populations (Figure 2C). These adaptations may change proliferation, metastatic potential or drug resistance, for example. It is also possible that evolution could generate novel clones with self-renewal potential, providing a rather more ‘hard-wired’, albeit evolving, route to the development of CSCs than does the process of interconvertibility described above. Both of these processes could be accommodated by the CSC model, as well as the hierarchical and stochastic models of heterogeneity.

However, although biological plasticity remains theoretical, the process of clonal evolution has recently been elegantly shown in primary and relapsed leukaemias by Mel Greaves’ group in London (Greaves, 2010), albeit at the level of a limited number of known targets. Using multi-plexed FISH analysis, Greaves and co-workers have identified a progressive accumulation of up to eight genetic changes in single cells. From this they have been able to determine the complex clonal architecture of individual leukaemias, showing the process of clonal evolution.

## The first identification of CSCs

Having identified the possibility of a cell population with the ability to initiate an entire ‘tumour’, the next major step in CSC biology was to identify that population. It was with the development of fluorescent antibodies, flow cytometry and associated cell sorting, that the reproducible isolation of phenotypically defined cell populations became possible. Furthermore, the development of mouse strains with profound immunodeficiencies enhanced the transplantation of malignancy. With these developing tools, pioneering work from John Dick's laboratory in Toronto set what remains the standard for identification of CSCs. Dick and colleagues showed that, in human acute myeloid leukaemia (AML), a rare malignant cell with the ability to repopulate the entire original disease over several transplantations, implying self-renewal and capacity to differentiate, was only found within the immature CD34^+^CD38^−^, and not the CD34^+^CD38^+^, sub-population (Lapidot *et al*, 1994; Bonnet and Dick, 1997). Blasts with potential to engraft the recipient immunodeficient mice were identified with a frequency of between 0.2 and 200 per million unsorted mononuclear cells, but in the CD34^+^CD38^−^ sub-population, their frequency rose to between 1 in 100 000 and 1 in 40.

The CD34^+^CD38^−^ immunophenotype is similar to that of normal HSCs, providing the intriguing possibility that this normal population might be the cell population in which the disease arises. Subsequent work using colony-forming assays and lentiviral vector tracking identified not only cells able to repopulate the disease over several transplantations, but cells repopulating only over a single transplantation as well as populations of apparently quiescent stem cells which appeared only after serial transplantation (Hope *et al*, 2004). This hierarchy closely mimicked the normal process of haematopoietic precursor development, in which increasing differentiation is accompanied by diminishing self-renewal, and suggesting that the hierarchy model of tumour heterogeneity might explain the CSC biology of childhood AML.

## Cancer stem cells in childhood acute lymphoblastic leukaemia (ALL)

The situation in childhood B cell precursor ALL has been less straightforward to define. Early studies showed that in both high and standard risk leukaemias, as with AML, populations with the HSC-like immunophenotype CD34^+^CD19^−^ contained the only source of ALL stem cells (Cobaleda *et al*, 2000; Cox *et al*, 2004). Subsequent studies have failed to confirm these findings. Indeed two groups have shown that in both the high risk Philadelphia chromosome positive and standard risk TEL/AML1-positive ALL, the B cell restricted population, expressing the B lymphoid differentiation marker CD19, is the only one to harbour ALL stem cells (Castor *et al*, 2005; Hong *et al*, 2008).

Recently, work from our own laboratory and others’, has shown that, as with AML, it is possible to isolate malignant populations with phenotypes corresponding to all normal developing B cell precursors including, in high risk disease, those with HSC phenotype CD34^+^CD19^−^ (Castor *et al*, 2005; Hotfilder *et al*, 2005). In contrast to AML, however, self-renewal, as shown by serial transplantation in immunodeficient mice, is not restricted to the HSC-like CD34^+^CD19^−^ population, but is found in populations corresponding to a range of normal B precursor populations (Kong *et al*, 2008; le Viseur *et al*, 2008). Indeed, we have shown that cells from the immunophenotypically most ‘mature’ population, CD34^−^CD19^+^, were able to recapitulate the entire disease phenotype, including the most ‘immature’ CD34^+^CD19^−^ blasts. We were also able to show that transcriptional differences exist between the blast populations, with those showing a more mature cell surface immunophenotype, also transcribing developmentally appropriate genes including late B cell transcription factors and immunoglobulin gene products. The debate continues, however, over how best to explain the differences between these most recent findings and those of earlier studies, but the hierarchy model, which seems to describe AML so faithfully, does not seems to be suitable in B precursor ALL.

## Solid tumour CSCs

As with B precursor ALL, the biology of CSCs in solid malignancies remains largely undefined. The first solid CSCs were identified in breast tumours in 2003 (Al-Hajj *et al*, 2003), since when CSCs have been isolated from brain (Hemmati *et al*, 2003; Singh *et al*, 2003), colon (O’Brien *et al*, 2007; Ricci-Vitiani *et al*, 2007), melanoma (Fang *et al*, 2005), pancreatic (Hermann *et al*, 2007; Li *et al*, 2007), prostate (Collins *et al*, 2005), ovarian (Bapat *et al*, 2005; Alvero *et al*, 2009), hepatic (Ma *et al*, 2007), lung (Ho *et al*, 2007; Eramo *et al*, 2008) and gastric cancers (Fukuda *et al*, 2009; Takaishi *et al*, 2009). Progress, however, has been complicated by the lack of clearly defined developmental surface markers specific for individual tumour types. Instead, isolation of many solid CSCs has been carried out using a number of adhesion markers including CD44 and CD24, or direct or indirect evidence of multidrug efflux proteins including ABCB5. CD133 (Prominin1), an apical plasma membrane protein found predominantly on embryonal epithelial structures, was used to isolate neural CSCs from a range of paediatric brain tumours (Singh *et al*, 2003). CD133 is expressed in many different types of stem cells and is thought to be involved in the attachment of stem cells to their niche. It provided the first solid tumour CSC marker relating to the stem cell phenotype of the host tissue. Since then, CD133 has continued to identify tumour cells with self-renewal capacity in a number of other solid malignancies, although there is ongoing debate as to quite how universal a marker it provides within the solid tumour CSC field (Wu and Wu, 2009).

To complicate the matter further, there have, as in childhood ALL, been a number of conflicting findings, with different groups isolating CSCs from differing, and occasionally ‘opposing’ cell fractions. Two principle theories exist to explain these variations. The first is that histologically similar tumours may have differing biology and that this is reflected in both the phenotype of CSCs and their presence or absence in a particular tumour. Alternatively, variations in experimental design may account for the conflicting findings in both haematological and solid tumour CSC research.

The biology of these CSC populations and, importantly, the niche specific to each of them, is the object of much ongoing research. Although a comprehensive review of the field is beyond the scope of this minireview, we would like to briefly mention an important biological area, that of epithelial-to-mesenchymal transition (EMT). Epithelial-to-mesenchymal transition is the transformation of highly ordered, communicating, epithelial cells to rather less organised mesenchymal cells, with the capacity to survive without cell–cell adhesion, to migrate and invade neighbouring tissues. Along with its corollary, mesenchymal-to-epithelial transition, EMT is an essential component of normal embryological development. Increasing interest in EMT has shown that this process may confer these key ‘malignant’ properties on cancer cells. Moreover, immune-mediated induction of EMT in epithelial breast cancer cells resulted in a mesenchymal population with the breast cancer CSC immunophenotype CD24^−/lo^CD44^+^ as well as the capacity to repopulate the malignancy after transplantation of low cell numbers. This was in contrast to the original epithelial cell type which required 1000-fold higher cell dose to initiate a malignancy (Santisteban *et al*, 2009). Epithelial-to-mesenchymal transition and its relevance to malignancy has recently been reviewed in detail (see Thiery *et al*, 2009) and may prove to be a key biological process in epithelial CSC biology.

## The importance of the experimental assay

Several recent publications have challenged the frequent assertion that CSCs are necessarily a rare phenomenon, by showing that assay conditions can have a significant effect on the engraftment of transplanted malignancies. Limitations on the ability of recipient microenvironmental/niche factors to successfully provide the survival and growth signals required to support engraftment are compounded by damage to cells during isolation and preparation, the effect of residual recipient immunity and, in haematological malignancies, a lack of homing factors to allow leukaemic stem cells to engraft a suitable bone marrow niche environment.

The development of mouse strains more heavily immunosuppressed than the scid and NOD/scid mice used in early AML studies has been a major step forward. NOD/scid mice with additional knock out of the IL2-R *γ* chain (NSG and NOG mice) lack all B, T and NK cells and have deficiencies in macrophage and complement function and are the current gold standard species. The enhanced immunosuppression is believed to result in improved levels of engraftment and consequent increase in CSC prevalence. However, recent work looking at the effects of residual immune function on clearance of antibody-labelled cells, has shown that even these most immunosuppressed species are able to clear both normal and malignant cells transplanted intravenously, thus reducing engraftment, although to a lesser extent than traditional NOD/scid mice (Taussig *et al*, 2008). Of particular note is the ability of residual immune function to clear AML blasts labelled with certain anti-CD38 antibodies, commonly used for immunophenotypic sorting, particularly in AML stem cell research. This Fc receptor-mediated clearance can be reduced by further immunosuppression with either IVIG or the anti-IL2 receptor antibody, anti-CD122, or by direct injection into the bone marrow. With these modifications, our group and others (data from Curt Civin's and Jean-Pierre Bourquin's laboratories) have presented data at recent meetings of the American Society of Haematology showing xeno-engraftment of ALL with as few as 10–100 transplanted cells.

Similar experimental caveats need also to be considered in the solid tumour CSC field. The melanoma stem cell, initially shown to be rare with a frequency of 1 in 10^6^ cells using a NOD/scid xenograft model, was found to be as frequent as 1 in 4 with the use of the NSG mouse and an experimentally enhanced host microenvironment (Quintana *et al*, 2008). Additional improvements in transplantation techniques such as orthotopic transplantation and co-engraftment with human stromal cells or artificial supporting matrices are increasingly being used to improve the sensitivity and clinical accuracy of the immunodeficient mouse model. Furthermore, the development of *in vivo* bioluminescent/fluorescent imaging holds great promise for the real time, *in vivo* monitoring of disease spread and response to therapy (Chanda *et al*, 2009; Kondo *et al*, 2009).

## Genetic pathways and the biology of CSCs

Understanding the genetic basis for cancer development is an important step in the development of novel therapies targeting the CSC. Numerous genes and signalling pathways connected with stem cell biology have been identified as important in cancer biology. Amongst others, *NOTCH*, *HOX* genes, *STAT5*, *SHH*, *FLT3*, *PI3K/AKT/mTOR/NF-κB* and telomerase have all been reported. An example of such a pathway is centred around *BMI1*. *BMI1* is a Polycomb group protein, which together with Ring1 proteins, is part of PRC1 complex that has histone H2A-K119 ubiquitin E3 ligase activity. *BMI1* has a role in *HOX* gene (*HOXC13*) silencing by H2A ubiquitylation (Cao *et al*, 2005). *BMI1* is also known to be important in the regulation and maintenance of proliferative/self-renewal potential in both normal haematopoietic and leukaemic stem cells (Park *et al*, 2003). Upon knockdown of *BMI-1,* cells lose their ability to engraft and reconstitute leukaemia in mice (Lessard and Sauvageau, 2003).

Another pathway altered in multiple malignancies is the WNT signalling pathway. WNT is a group of secreted signalling proteins that bind receptor molecules (e.g., Frizzled) on the surface of target cells. Downstream signalling is mediated by several transducing proteins (e.g., *β*-catenin) to activate its target genes, which include *MYC* or *CCND1* (cyclin D1). Interestingly, WNT can be interlinked with, as well as converge on, other pathways to activate similar targets. The strongest evidence of the importance of the WNT pathway to CSC biology has been reported in myeloid leukaemias. Zhao *et al* (2007) have shown the necessity of *β*-catenin for self-renewal of both normal hematopoietic stem cells and CSCs in chronic myeloid leukaemia in a mouse model, whereas more recently, Wang *et al* (2010) showed that *β*-catenin activation is necessary for myeloid precursor transformation in a *HoxA9/Meis1*-transduced model of AML. The WNT signalling pathway has also been reported to be altered in classical medulloblastoma arising from ventricular zone stem or progenitor cells, whereas in medulloblastomas arising from the external germinal layer, it is the Hedgehog pathway which is activated (for a review see de Bont *et al*, 2008).

Critical to our understanding of CSC biology is understanding the control of the principle stem cell property – self-renewal. One exciting source of information on self-renewal is leukaemias characterised by fusion genes. A substantial proportion of leukaemias result from one of a large number of fusion genes, some of which are sufficient either, in the case of *TEL/AML1*, to initiate a ‘preleukaemic stem cell’ phenotype with the ability to self-renew (Hong *et al*, 2008), or to initiate frank myeloid malignancy in the case of *MLL/ENL* and *MOZ/TIF2* (Cozzio *et al*, 2003; Huntly *et al*, 2004). The ability of some fusion genes to drive malignant transformation, and therefore presumably to initiate the self-renewal programme, has made them ideal candidates for further study.

By creating an *Mll/AF9* knock-in model of AML, in which the fusion gene remained under endogenous promoter control, John Kersey's group in Minnesota demonstrated that transformation occurred only in hematopoietic stem cells and not committed granulocyte-monocyte precursors as can be achieved with the higher expression levels resulting from retroviral transduction (Chen *et al*, 2008). This group showed the upregulation of a programme of genes involved in stem cell biology including several *Hox* genes and *Meis1*, well-characterised targets of MLL fusion proteins.

More recently, a retroviral transduction model of a number of MLL fusion genes has been used to identify the transcriptional programme responsible for the maintenance of a self-renewing phenotype (Somervaille *et al*, 2009). Under these expression conditions, this group defined a leukaemic stem cell maintenance programme, containing some 560 genes, based on the positive or negative correlation of gene expression with CSC frequency. This CSC maintenance programme resembles the committed myeloid progenitor programme more closely than the HCS programme, but shares similarities with embryonic stem cells. This programme is shared with a number of other, poor prognosis, malignancies.

Finally, a novel mechanism for identifying the biology of stem cell self-renewal and differentiation has very recently been described. The induction of pluripotency in CSCs may allow further analysis of mechanisms able to control these critical pathways including, for example, modification of epigenetic codes (Miyoshi *et al*, 2010).

## Implications for cancer therapeutics

To cause relapse, CSCs must have survived primary treatment. A number of factors may underlie this, including stem cell quiescence, protected niche environment, upregulated expression of xenobiotic efflux pumps, enhanced anti-apoptotic and DNA repair pathways as well as other survival mechanisms.

The relevance of these properties to stem cell survival is exemplified by the effects of the tyrosine kinase inhibitor imatinib mesylate in chronic myeloid leukaemia (CML). Chronic myeloid leukaemia is a stem cell disease par excellence – a rare population of cells with a HSC-like phenotype are able to self-renew and differentiate to form all haematopoietic lineages which therefore harbour the definitive genetic aberration, t(9;22)(q34;q11) – the Philadelphia chromosome. The fusion product, a tyrosine kinase, is sufficient to initiate CML. Despite the immense success of Imatinib and subsequent tyrosine kinase inhibitors, in controlling disease bulk, a rare population of quiescent stem cells remains inherently resistant to this therapy (for review see Elrick *et al*, 2005). A novel strategy for gaining therapeutic access to these quiescent cells using histone deacetylase inhibitors in combination with Imatinib has recently been described (Zhang *et al*, 2010) and, together with increased understanding of the disease sensitivity to Imatinib therapy in the clinical setting may lead to an improved disease control in CML.

A number of targets with enhanced activity in CSCs have been identified and investigated therapeutically. The Parthenolide analogue DMAPT, has been shown in AML. This potent inhibitor of NF-κB, which is highly active in AML stem cells but not normal HSCs, results in apoptosis of both AML and CML blast crisis stem cells, sparing normal HSCs (Guzman *et al*, 2005). Another example of CSC-specific targeting is the mTOR inhibitor Rapamicin (Yilmaz *et al*, 2006), whereas hTERT has been identified as a potential target in high-risk infant ALL with the translocation t(4;11)(q23;q23) (Gessner *et al*, 2008).

One alternative solution is to target oncogenic fusion genes, their transcripts or protein products directly where they exist. Clearly the fusion gene should not be possessed by normal stem cells, making it an attractive therapeutic target. This approach has, to date, been hampered by difficulties in the delivery of therapies, particularly those targeting the fusion transcript by RNA interference techniques.

Finally, as the importance of the stem cell niche becomes better understood, targeting this element of the CSC's biology may prove possible. Once again, drawing parallels with the well-defined HSC niche has allowed our understanding of leukaemic stem cell-niche biology to develop rapidly. Potential targets, including NOTCH and WNT pathways, the chemokine receptor CXCR4 and adhesion molecules, are all likely to have a role in the leukaemic stem cell microenvironment provided by the bone marrow niche (reviewed in Lane *et al*, 2009). Outside of haematological malignancies, the importance of the vascular niche has received most attention. Vascular recruitment is essential to solid tumour development and clinical trials of vascular endothelial growth factor receptor antagonists are providing positive results. A body of research now supports the importance of the vascular niche to the support of brain tumour stem cells, raising the possibility that anti-vascular drugs may be used to disrupt vascular niche–CSC interactions (reviewed in Ghotra *et al*, 2009).

The clinical relevance of CSCs has yet to be shown. It is widely believed that in order to prevent relapse, efficiently targeting the CSC is an essential objective. If achieved selectively, novel CSC-targeting drugs may also reduce the toxicity associated with current, unselective anti-proliferative chemotherapy.

## Conclusion

The concept of tumour heterogeneity, as well as the belief that ‘only a small fraction of the tumour cells have the power of proliferation, or that every cell with such power is subjected to considerable hazards in the process of being transplanted’ (Hewitt, 1953), has been known for many decades. However, the last 15 years has seen rapid progress in the identification and isolation of CSCs.

The xenotransplantation model, developed in John Dick's laboratory, has been successfully extended to CSC research in lymphoblastic leukaemia, as well as a growing number of solid malignancies. However, many challenges remain, including the universal adoption of the most sensitive self-renewal assays to provide consistent and accurate results. An increasing body of CSC evidence may confirm that a stem cell hierarchy is not applicable to all tumours, whereas the CSC may turn out to be more common than initially believed, at least in some malignancies. Indeed, the absolute frequency may depend not only on the tumour type or cell of origin, but on the specific oncogenic changes driving a malignancy's ‘stemness’ (Heuser *et al*, 2009). Furthermore, integrating processes such as plasticity, interconvertibility and clonal evolution in a tumour-specific manner will lead to substantially greater complexity in the CSC model. However this will be critical, as to focus on one stem cell theory at the expense of others risks discounting biology with therapeutic potential, in favour of creating a concise model.

The ultimate challenge in coming years will be understanding the stem cell ‘programme’, particularly the control of self-renewal, in an attempt to develop novel, stem cell-directed therapies. An improved understanding of clonal evolution will be critical if we are to ensure that cancers are not able to evolve mechanisms to evade our new directed therapies. However, reducing the risk of relapse and minimising long-term side effects for our patients should always remain the ultimate goal of understanding the CSC.

## Figures and Tables

**Figure 1:**
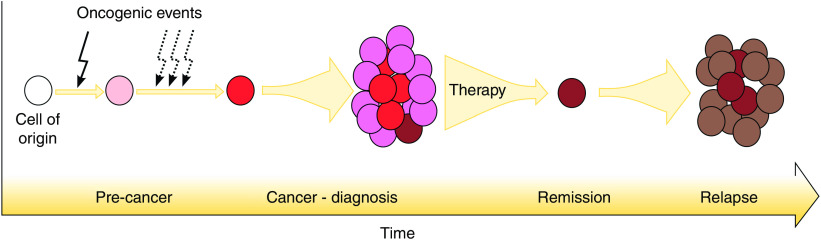
The cancer stem cell theory of tumour development and relapse initiation. An initial oncogenic event (solid arrow) occurring in a normal cell may create a precancerous cell, or directly result in malignant transformation. The oncogenic event is likely to require a number of supporting genetic/epigenetic events (hashed arrows). By the point of clinical diagnosis, the heterogeneous tumour contains cells which have, or are able to activate their stem cell programme and may be able to evade standard therapy. Any cancer stem cells evading therapy are able to divide and differentiate to repopulate the tumour.
